# Study on the Effect of PVAc and Styrene on the Properties and Microstructure of MMA-Based Repair Material for Concrete

**DOI:** 10.3390/ma16113984

**Published:** 2023-05-26

**Authors:** Zemeng Guo, Lingling Xu, Shijian Lu, Luchao Yan, Zhipeng Zhu, Yang Wang

**Affiliations:** College of Materials Science and Engineering, Nanjing Tech University, Nanjing 211816, China; 202061103028@njtech.edu.cn (Z.G.); 202161103065@njtech.edu.cn (S.L.); 202161203204@njtech.edu.cn (L.Y.); 202161103006@njtech.edu.cn (Z.Z.); 202261103012@njtech.edu.cn (Y.W.)

**Keywords:** methyl methacrylate, concrete repair material, low shrinkage, shrinkage stress, mechanical properties

## Abstract

Methyl methacrylate (MMA) material is considered to be a suitable material for repairing concrete crack, provided that its large volume shrinkage during polymerization is resolved. This study was dedicated to investigating the effect of low shrinkage additives polyvinyl acetate and styrene (PVAc + styrene) on properties of the repair material and further proposes the shrinkage reduction mechanism based on the data of FTIR spectra, DSC testing and SEM micrographs. The results showed that PVAc + styrene delayed the gel point during the polymerization, and the formation of two-phase structure and micropores compensated for the volume shrinkage of the material. When the proportion of PVAc + styrene was 12%, the volume shrinkage could be as low as 4.78%, and the shrinkage stress was reduced by 87.4%. PVAc + styrene improved the bending strength and fracture toughness of most ratios investigated in this study. When 12% PVAc + styrene was added, the 28 d flexural strength and fracture toughness of MMA-based repair material were 28.04 MPa and 92.18%, respectively. After long-term curing, the repair material added with 12% PVAc + styrene showed a good adhesion to the substrate, with a bonding strength greater than 4.1 MPa and the fracture surface appearing at the substrate after the bonding experiment. This work contributes to the obtaining of a MMA-based repair material with low shrinkage, while its viscosity and other properties also can meet the requirements for repairing microcracks.

## 1. Introduction

Concrete is one of the most commonly used building materials in the world, but cracks are always present in concrete owing to its shrinkage, external freeze-thaw, chemical erosion and other processes [1,2]. They can destroy the strength of the concrete and shorten its service life, and can even cause further structural damage if left untreated [3]. Therefore, it is necessary to repair cracks of concrete in time, whether from the perspective of economy or environmental protection [4].

At present, repair materials commonly used in the world can be divided into three types [5]: inorganic repair material, which is the most widely used material to repair cracks in the world, mainly including sulphoaluminate cement, magnesium phosphate cement and expansive cement [6,7,8]; organic repair material, which usually refers to polymer resins such as polyurethane, epoxy and acrylate [9,10,11]; and organic–inorganic composite repair material, which combines the advantages of organic material and inorganic material in the form of polymer-modified mortar/concrete, polymer-impregnated mortar/concrete and polymer mortar/concrete [12,13,14].

Grouting is a method that can be used to fill the cracks with appropriate seriflux to achieve the purpose of reinforcement and antiseepage, and it is widely used in the repair works of microcracks [15,16]. Penetration capability is an important evaluation criterion for the success of grouting; especially for those microcracks with a width less than 0.5 mm [17], the grouting performance of cement-based repair materials and organic–inorganic composite repair materials is very limited due to their particle size [18,19], while the organic repair materials with excellent fluidity are the best repair materials for microcracks [20]. Epoxy resin is a typical organic repair material, with high strength, excellent chemical resistance and good adhesive property. However, its relatively high viscosity makes it limited in repairing microcracks [21]. Furfural/acetone dilution system is usually used to reduce the viscosity of epoxy resin, but furfural is toxic and volatile, which will cause harm to human body and environment [22]. Its high brittleness and poor toughness also greatly limit its application [23]. Polyurethane is also a commonly used chemical grouting material, which has good impermeability and high strength [24]. However, the curing process of polyurethane requires reaction with water, so polyurethane is mostly used in the repair of water conservancy engineering [25]. In addition, polyurethane also needs diluent to reduce its viscosity [26]. Therefore, it is very important to develop an organic grouting material with strong permeability and excellent mechanical properties.

The viscosity of methyl methacrylate (MMA)-based repair material can be as low as 0.8 mPa·s, and it is even suitable for repairing microcracks with a 0.05 mm width due to its good fluidity [27,28]. Moreover, it has good chemical resistance and strong adhesion with old concrete [29]. However, the biggest disadvantage of MMA-based repair material is its high shrinkage during polymerization, which can reach about 21%; this disadvantage limits its application in repairing engineering to a great extent [30]. The main cause of volume shrinkage is the transformation of van der Waals forces between MMA monomers into covalent bonds after polymerization [31]. This results in a smaller molecular distance and thus tighter arrangement, and creates shrinkage stress that would affect the bonding effect, so it is vital to reduce its volume shrinkage [32,33]. Common methods to effectively reduce polymerization shrinkage include (1) synthesizing low-shrinkage resin, (2) adding inorganic filler and (3) adding low-shrinkage additives [34,35,36,37]. A vast number of studies on the reduction of volume shrinkage of MMA have been carried out, but there remains no guarantee that the viscosity can meet the requirements for repairing microcracks. Han [38] found that after adding perchloroethylene, the shrinkage of the MMA repair material decreased to a certain extent and then decreased significantly after the further addition of inorganic filler calcium carbonate, down to about 7%. However, the addition of calcium carbonate led to an increase in the viscosity of the resin, which prevented the calcium carbonate from being well dispersed in the system and thus reduced the bending strength of the material. Wang [39] used epoxy resin to modify the MMA material, with the results showing that the bonding strength of the repair material increased, but the viscosity also increased considerably, with continual addition of diluent being needed. Wu [40] synthesized MMA, epoxy and polyurethane prepolymer by using the interpenetrate polymer network technique. Polyurethane improved the volume stability and the flexibility but also had a significant impact on the viscosity of the system. Compared with other repair materials, MMA possesses a low viscosity as its unique advantage, so it is important to pay attention to its viscosity change while modifying it. In order to repair microcracks, in addition to maintaining good fluidity and high mechanical properties, the high shrinkage of MMA-based repair material also needs to be reduced. The addition of low-shrinkage additives can effectively reduce the shrinkage of the resin. Styrene is a nonpolar low-shrinkage additive that can form a two-phase system with resin, thereby reducing the shrinkage. The effect of reducing shrinkage is general, but its mechanical strength is relatively high. Polyvinyl acetate (PVAc) is a polar-low shrinkage additive that forms a homogeneous system with resin before curing and evenly separates phases after curing. It has an excellent shrinkage reduction effect but poor mechanical strength. PVAc + styrene low shrinkage additives have good overall performance in terms of shrinkage and mechanical strength.

In this study, in order to obtain a repair material that meets the requirements for repairing microcracks, the effects of PVAc + styrene as low-shrinkage additives on the shrinkage, viscosity, bond strength, bending strength and tensile strength of MMA-based repair material were investigated. The modification mechanism of low-shrinkage additives was also studied using SEM, FTIR and DSC.

## 2. Materials and Experiments

### 2.1. Raw Materials

Methyl methacrylate (MMA), the initiator benzoyl peroxide (BPO), the plasticizer dioctyl phthalate (DOP), the accelerator N-N-dimethylaniline (DMA) and styrene were all supplied by Yonghua Chemistry Co., Ltd. (Suzhou, China). Polyvinyl acetate (PVAc) was provided by Shanghai Meryer Chemical Technology Co., Ltd. (Shanghai, China).

### 2.2. Specimen Preparation

In order to ensure the normal progress of polymerization, it is necessary to remove the polymerization inhibitor which is added to MMA during the storage process. Although the content is relatively low, generally not more than 0.001%, this can also have an impact on the polymerization reaction. Therefore, the following method was used to remove the inhibitor before polymerization: MMA monomer was subjected to open distillation for 10 min in a water bath at 50 °C.

Table 1 shows the raw material ratio of the MMA-based repair material. The mass ratio of PVAc to styrene was 7:3, and PVAc was poured into styrene to dissolve. The mass ratio of MMA: BPO: DOP: DMA was 100:0.6:30:0.6. After weighing was completed, MMA, PVAc + styrene, BPO and DOP were added together into a three-mouth flask and then stirred constantly in a water bath at 80 °C. During the reaction process, in order to prevent the explosive polymerization phenomenon, it was important to be clear about the change of viscosity of the solution. If the viscosity of the system was found to be large, the three-mouth flask would be immediately removed from the water bath and cooled down.

### 2.3. Testing Method

#### 2.3.1. Shrinkage

The type of shrinkage of MMA-based repair material measured in this experiment was chemical shrinkage, which was tested using the following method: first, the volume of test tube V_1_ was obtained using the mass and density of the distilled water. Then, the repair material prepared in Section 2.2 that was mixed with DMA was used to fill the test tube and was cured at room temperature. Therefore, the volume of the specimen before curing was *V*_1_, and the mass of the specimen before curing was determined from the difference in the mass of the test tube before and after the addition of the repair material and noted as *m*_1_. After the specimen was cured, the test tube was broken, the specimen after curing was weighed to obtain the mass *m*_2_, and then the volume *V*_2_ of the cured specimen was measured using the drainage method. The chemical volume shrinkage was calculated as shown in Equation (1).

(1) $$S=\frac{\frac{m_{2}}{V_{2}}-\frac{m_{1}}{V_{1}}}{\frac{m_{1}}{V_{1}}}\times 100\%$$

where *S* is the volume shrinkage of the specimen (%); *m*_1_ and *m*_2_ are the mass of the specimen before and after curing (g), respectively; and *V*_1_ and *V*_2_ are the volume of the specimen before and after curing (mL), respectively.

#### 2.3.2. Shrinkage Stress

The rotation rheometer of Anton Paar MCR 302 was used to test the change of internal stress of the polymer during curing under the controlled strain mode with a strain value of 1% and a frequency of 1 Hz.

#### 2.3.3. Viscosity

The pointer type NDJ-1 rotary viscometer was used to measure the viscosity of the repair material. First, the repair material was placed in a beaker, and then the appropriate rotor and rotational speed were selected according to the viscosity of the solution. Finally, the rotor was immersed vertically in the solution, the starting switch was pressed, and the data were read after the indicator was stable.

#### 2.3.4. Bond Strength

**Figure 1:**
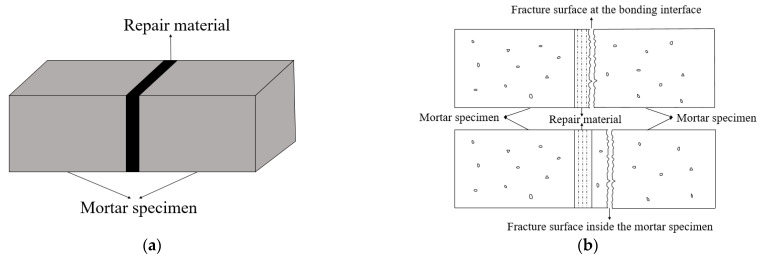
(**a**) The repair model and (**b**) two types of fracture surfaces.

Ordinary Portland Cement mortar (cement:sand:water = 1:3:0.5 by weight) was used to test the bond strength. When OPC mortar was cured for 28 d under standard curing conditions (at 20 ± 2 °C and RH 95%), it was first fractured under the flexural strength test using the universal testing machine (ETM-F), and then the distance between the two fractured specimens was kept at 3 mm and pasted with tape. After the gap was filled with MMA-based repair material, the specimen was put into an oven at 40 °C for 4 h and then placed in a standard curing box; the repair model is shown in Figure 1a. The flexural strength was used as an index to measure the bond strength of the repair material and the OPC mortar. As shown in Figure 1b, if the fracture surface of the specimen appears at the bonding interface, the obtained flexural strength value is the bond strength, and if the fracture surface occurs inside the mortar specimen, the bond strength of the repair material and the OPC mortar is greater than the obtained flexural strength.

#### 2.3.5. Bending Strength

After accelerator DMA was added to the prepared repair material, the material was poured into a mold with dimensions of 100 mm × 15 mm × 5 mm, and finally the maximum load of bending failure of the specimen was recorded by using the strength testing machine. The bending strength of the repair material was calculated according to Equation (2):

(2) $$R_{f}=\frac{{3F}_{f}L_{f}}{2{b_{f}h_{f}}^{2}}$$

where *R_f_* is the bending strength of the repair material (MPa); *F_f_* is the maximum load of bending failure of the repair material (N); *L_f_* is the distance between two points of the repair material under force (mm); and *b_f_* and *h_f_* are the width and height of a cross-section of repair material (mm), respectively.

#### 2.3.6. Tensile Strength

After the repair material was poured into a mold with a size of 40 mm × 10 mm × 2 mm, tensile test was carried out on a strength testing machine. The tensile load was applied along the length of the specimen at a rate of 1 mm/min until the tensile failure of the specimen. The tensile stress–strain curve was plotted, from which the tensile strength and elongation at break of the material could be discerned. The tensile strength and elongation at break of the material were calculated according to Equations (3) and (4):

(3) $$\sigma_{t}=\frac{F_{t}}{b_{t}\cdot h_{t}}\times 100\%$$

where *σ_t_* is the tensile strength of the material (MPa); *F_t_* is the maximum load of tensile failure of the specimen (N); and *b_t_* and *h_t_* are the width and height of a cross-section of specimen fracture (mm), respectively.

(4) $$\varepsilon_{t}=\frac{G_{t1}-G_{t0}}{G_{t0}}\times 100\%$$

where *ε_t_* is the elongation at a break of the specimen (%), *G_t_*_1_ is the initial marking distance of the specimen (mm), and *G_t_*_0_ is the marking distance where the specimen was fractured(mm).

#### 2.3.7. Microscopic Characteristics and Morphology

The molecular structure of the repair material were determined with an infrared spectrometer (Nicollet Nexus 670, Shanghai, China), and the polymerization between the MMA monomer and other additives was studied by analyzing the functional groups. The glass transition temperature (T_g_) of the polymer was determined with a differential scanning calorimeter (DSC 404 F1 Pegasus^®^, Shanghai, China). The microscopic morphology of the polymerization product was observed using SEM (JSM-6510, Shanghai, China).

## 3. Results and Discussion

### 3.1. Effect of PVAc + Styrene on Chemical Shrinkage of MMA-Based Repair Material

When no admixture was added, the chemical volume shrinkage of MMA-based concrete repair material was as high as 21%, which would definitely affect its use in repair engineering. The volume shrinkage of the material will create stress toward the interior of the material at the bonding interface, which may lead to the failure of the interface bonding or even secondary cracking [41]. Theoretically, the higher the chemical volume shrinkage of a material is, the higher its shrinkage stress, so it is necessary to add low 0hrinkage additives to reduce its shrinkage. The PVAc + styrene solution was added into MMA to prepare the repair material, and the variation law of chemical shrinkage of MMA-based repair material with different contents of PVAc + styrene was investigated, with test results being presented in Table 2.

As can be seen from the Table 2, after the addition of low-shrinkage additives, the chemical volume shrinkage and the shrinkage stress of the material decreased significantly with the increase of the proportion of additives; notably, when the proportion of PVAc + styrene was 12%, the chemical volume shrinkage was as low as 4.78%, and the shrinkage stress reduced from −5.08 N to −0.64 N, which decreased by 87.4% (negative stress values indicate shrinkage). After the addition of low-shrinkage additives, one of the reasons for the significant reduction in chemical shrinkage of the material was that polystyrene was formed in the polymerization process, and polystyrene is a nonpolar low-shrinkage additive, which can reduce the chemical shrinkage of MMA resin to a certain extent. When the proportion of PVAc + styrene exceeded 12%, the chemical shrinkage of the MMA-based repair material gradually increased with the increase in the proportion of additives. Previous study has shown that the low-shrinkage additives act only as inert materials at contents higher than the upper limit of the effective content [42]. However, compared with 22.67% of the blank specimen without additives, the chemical volume shrinkage of the repair material was still very low.

### 3.2. Effect of PVAc + Styrene on the Polymerization Process of MMA-Based Repair Material

**Figure 2:**
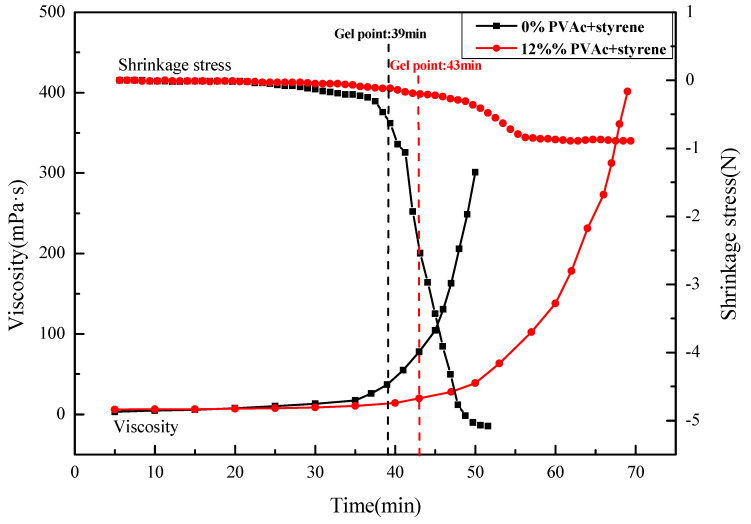
The curing process of MMA-based repair materials with different dosages of PVAc + styrene.

Viscosity is used to characterize the rheological property of the polymer, which varies with time [43]. A low viscosity of the polymer means that it has better fluidity and thus can penetrate into microcracks more easily. One of the most prominent advantages of MMA-based repair material compared with other repair materials is its low viscosity, which can repair microcracks smaller than 0.5 mm in width, so it is vital to test the change rule of the viscosity of MMA-based repair material in modification experiments. The effect of the reaction time on the viscosity of the MMA-based repair material was investigated by testing the viscosity of the repair materials with 0% and 12% PVAc + styrene, and the change in shrinkage stress during the polymerization was also measured to determining the reason for its reduction, with the results being shown in Figure 2.

The polymerization process of MMA, as shown in Figure 2, is similar to that reported in the previous research results, which can be basically divided into two stages, and the viscosity increases approximately exponentially with time [44,45]. Before gelation, the initiator decomposes into free radicals, so the viscosity almost remains constant. Then after gelation, with the increase in the number of free radicals and chain activation centers, the main chain of PMMA increases, and the viscosity of the system also changes significantly with reaction time. It should be noted that as the reaction proceeds, the viscosity increases sharply, making it difficult to remove the polymerization heat from the system, so it is necessary to control the reaction time to prevent explosion at this stage. The inflection point of the curve is considered to be the gel point. Since the movement of the macromolecules is relatively free before gelation, the shrinkage stress can be effectively released, while after gelation, the spatial network structure is formed, the movement of the molecular chains is difficult, and so the shrinkage stress gradually increases. Therefore, it can be seen that the shrinkage stress of the repair materials without and with 12% PVAc + styrene increases sharply after 39 min and 43 min of the gel point until reaching the maximum value, respectively.

In addition, by comparing the viscosity of MMA-based repair materials without PVAc + styrene and with 12% PVAc + styrene at the same time, we found that the gel point of the polymerization process was delayed after adding low-shrinkage additives. This is because with the addition of additives, the free radicals decomposed by the initiator activate not only the MMA molecules but also the styrene molecules. Therefore, the growth of the main chain of PMMA slows down, the rate of forming network structure decreases correspondingly, and the fluidity of the main chain increases, thus reducing the shrinkage stress. Therefore, the delay in the gel point retards the development of the stress and thus reduces the shrinkage of the material, which is similar to results reported elsewhere [46].

### 3.3. The Shrinkage Reduction Mechanism of PVAc + Styrene on MMA-Based Repair Material

In order to investigate the effect of PVAc + styrene on the structure of MMA-based repair material, MMA repair materials without and with 12% PVAc + styrene were tested using FTIR, DSC and SEM.

**Figure 3:**
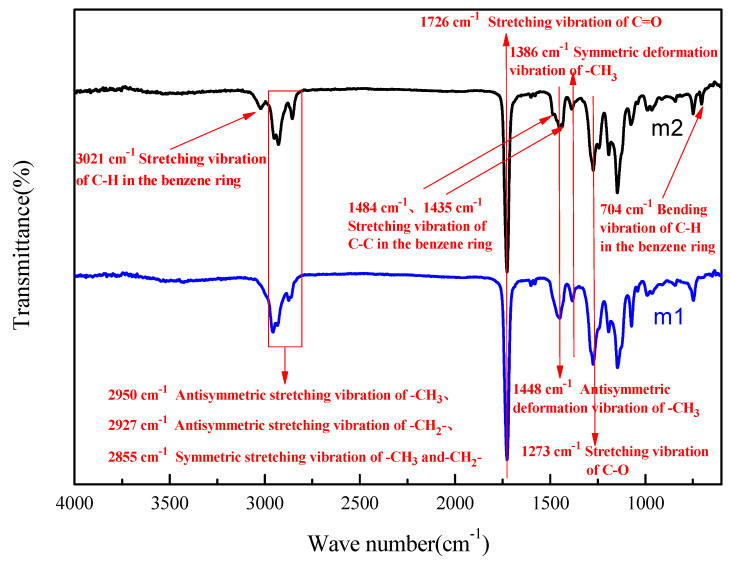
FTIR spectra of the polymerization products.of PS0(m1) and PS12(m2).

Figure 3 shows the FTIR spectra for the MMA-based repair materials with the ratios of PS0 and PS12 in the range of 500–4000 cm^−1^, with m1 being the PS0 repair material and m2 being the PS12 repair material. It can be noticed that there was no characteristic peak of C=C in either curve, indicating that during the reaction, both MMA and styrene underwent polymerization. In the m1 curve, the presence of the bands representing -CH_3_, -CH_2_-, C=O and C-O demonstrated the formation of polymethyl methacrylate. In the m2 curve, in addition to all the absorption bands of m1, the bands at 3021 cm^−1^ and 704 cm^−1^ were associated with the vibration of C-H in the benzene ring, the bands at 1484 cm^−1^ and 1435 cm^−1^ were attributed to the stretching vibration of C-C in the benzene ring, and these bands represented the formation of polystyrene.

**Figure 4:**
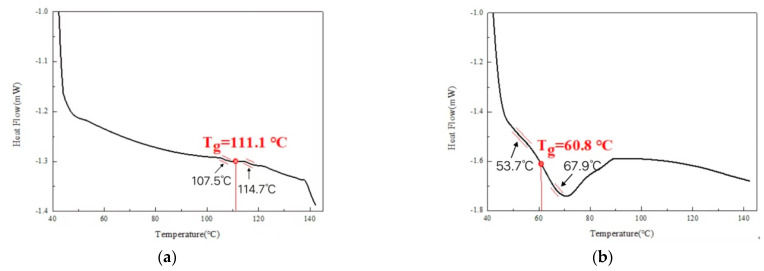
DSC curve of repair material (**a**) without and (**b**) with 12% PVAc + styrene.

Subsequently, for specifying whether the polymerization between MMA and styrene occurred, DSC analysis was carried out. MMA-based repair materials with and without 12% PVAc + styrene were ground into powder for DSC testing, and the obtained experimental results were shown in Figure 4.

As can be seen from the Figure 4, the glass transition temperature (T_g_) of the MMA-based repair material was about 111.1 °C without the low-shrinkage additives, while the T_g_ of the material decreased to 60.8 °C after the low-shrinkage additives were incorporated. This is mainly because after the addition of the low-shrinkage additives, the free radicals decomposed by the initiator activated not only the MMA molecules but also styrene molecules so that in the heating process, MMA and styrene in the system underwent copolymerization, which reduced the glass transition temperature.

**Figure 5:**
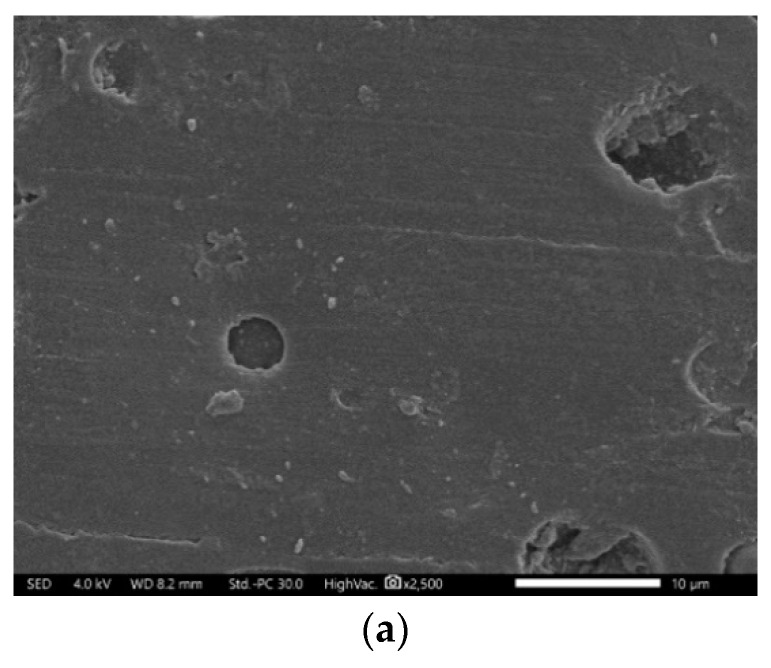

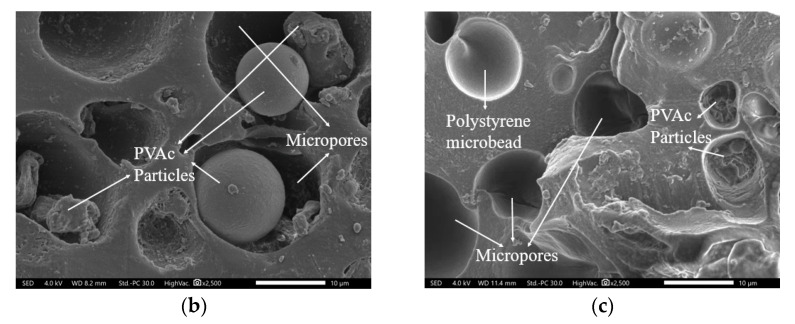
SEM micrographs of the polymerization products with different contents of PVAc + styrene. (**a**) No PVAc + styrene; (**b**,**c**) 12% PVAc + styrene.

The fractured surfaces of polymerization products with the ratios of PS0 and PS12 were also observed using SEM. In Figure 5a of, it can be seen from the specimen without additives that the overall morphology was a single-phase structure, dominated by the dense MMA continuous phase. As seen in Figure 5b,c with 12% PVAc + styrene, the fractured surface of the specimen showed a two-phase structure clearly, with the PVAc phase dispersed in the MMA phase in the form of particles. There were microvoids around the particles, which could compensate for the volume shrinkage to some extent. In addition, in Figure 5c, it can be seen that the polystyrene was unevenly dispersed in the MMA phase in the form of microbeads, which counteracted the volume shrinkage of the resin with its own sufficient expansion.

**Figure 6:**
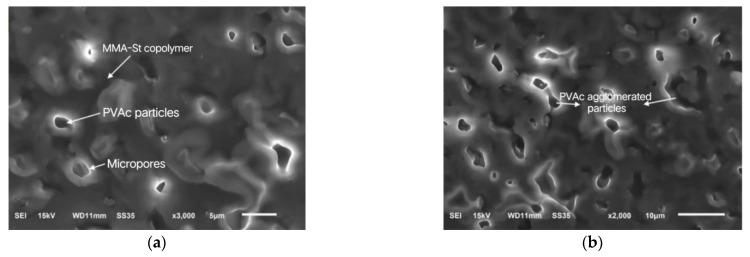
SEM micrographs of the polymerization products with different contents of PVAc + styrene. (**a**) 12% PVAc + styrene; (**b**) 14% PVAc + styrene.

As seen in Table 2, the shrinkage of MMA-based repair materials begins to decrease when the PVAc + styrene is greater than 12%. The reasons for the decrease were investigated, and the SEM of the polymerization products of the MMA-based repair materials of PS12 and PS14 is shown in Figure 6.

As can be seen from Figure 6a, when the dosage of PVAc + styrene was 12%, the PVAc particles were spherically dispersed in the repair material, with a size of about 1–3 μm, and there were micropores around them with a size less than 1 μm, which considerably reduced the shrinkage. When the dosage of PVAc + styrene increased to 14%, as shown in Figure 6b, the size of the PVAc particles became larger due to the agglomeration, showing lumpy and long stripes, and the size of some particles was even larger than 5 μm, which would affect its uniformity in the repair material and reduce the number and volume of micropores, making the effect of shrinkage reduction worse.

**Figure 7:**
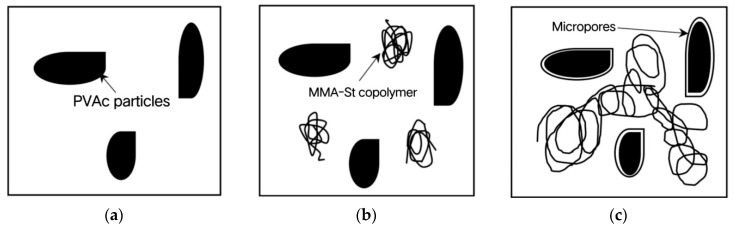
Mechanism of PVAc + styrene for MMA repair material. (**a**) Stage of phase separation; (**b**) Stage of macroscopic gel phase; (**c**) Stage of creating microspores and expanded.

From the above analysis of the copolymerization products, including FTIR spectra, DSC curves and SEM images, combined with the analysis on the shrinkage characteristics of unsaturated resins [47,48,49], the shrinkage reduction mechanism of this system can be deduced as follows:

1. Phase separation stage: PVAc is dissolved in styrene solution as sphere-like particles, and each particle occupies a certain volume. However, styrene is a nonpolar solvent, while PVAc is a polar polymer, which eventually leads to a decrease in the compatibility of PVAc with styrene, resulting in phase separation, which is shown schematically in Figure 7a.

2. Macroscopic gel phase stage: In the process of polymerization, when the temperature rises, the initiator BPO begins to decompose into free radicals, which undergo addition reactions with MMA or styrene. During this period, the polymerization reactions between MMA and MMA, between styrene and styrene and between MMA and styrene take place, forming a continuous phase of MMA, a dispersed phase of PVAc and a random copolymer of MMA-St within the system, which gradually produces a macroscopic gel as the degree of polymerization increases. The final structure of the copolymer surrounded by PVAc particles is shown in Figure 7b. In the newly formed structure, the benzene ring linked to the main chain of MMA produces repulsion and hindrance, forming a steric effect, which reduces the volume shrinkage to a certain extent.

3. Microspore creation and expansion stage: As shown in Figure 7c, when the reaction rate is about to reach its maximum, PVAc plays a role in accumulating styrene, as the heat formed by the polymerization reaction allows PVAc and unreacted styrene monomer to expand, compensating for the volume shrinkage. As the temperature of the system decreases, MMA phase and PVAc phase shrink at the same time. When the temperature is higher than T_g,PMMA_, the coefficients of thermal expansion of these two phases are basically the same. When the temperature is reduced to higher than T_g,PVAc_ and lower than T_g,PMMA_, the contraction effect of the PVAc phase is greater than that of the MMA phase, resulting in the formation of micropores at the interface between the two phases, and the micropores become larger and larger until the temperature is gradually reduced to T_g,PVAc_, (T_g,PVAc_ is 30 °C, T_g,PMMA_ is 105 °C). During the formation of micropores, the internal stress is partially released, so the shrinkage stress of the material is reduced after the addition of low-shrinkage additives.

### 3.4. Effect of PVAc + Styrene on the Mechanical Properties of MMA-Based Repair Material

Based on the results in Section 3.1, Section 3.2 and Section 3.3, it can be concluded that PVAc + styrene can improve the shrinkage of MMA-based repair material to a certain extent, but its effect on the mechanical properties of repair material still needs to be investigated. The interface of organic–inorganic composites is the main factor affecting their overall mechanical properties, so it is important to study the interfacial bonding property. The results of the bond strength of repair materials with different PVAc + styrene ratios at different ages are shown in the Table 3.

**Figure 8:**
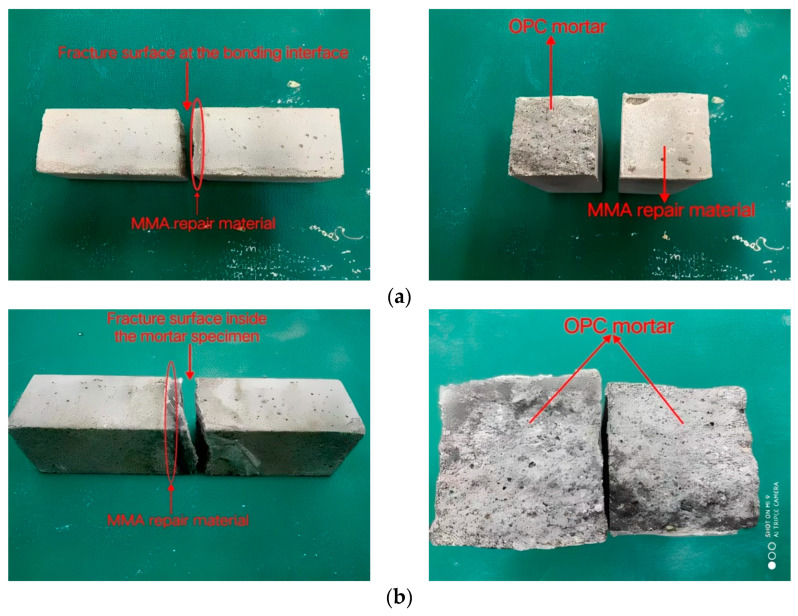
Two types of fracture surfaces: (**a**) fracture surface at the bonding interface and (**b**) fracture surface inside the OPC mortar.

As can be seen from the Table 3, the variation of bond strength at different ages with different proportions is basically the same. At 3 d, the bond strength of the repair material with low-shrinkage additives was relatively low, and all specimens fractured at the bonding interface. At 7 d, the bond strength was significantly higher than that at 3 d, and some specimens had fractures inside the OPC mortar. The specimens at 28 d all had fractures inside the OPC mortar, implying that the bond strength was greater than the strength value obtained, which was highly beneficial for the actual repair engineering. The two types of fracture surfaces of bonding specimens are shown in Figure 8. Figure 8a shows that the fracture surface appeared at the bonding interface between the repair material and the OPC mortar, indicating that the bonding surface was the weak link of the specimen. Figure 8b shows that the fracture surface occurred inside the OPC mortar, demonstrating a good interfacial bonding performance.

**Figure 9:**
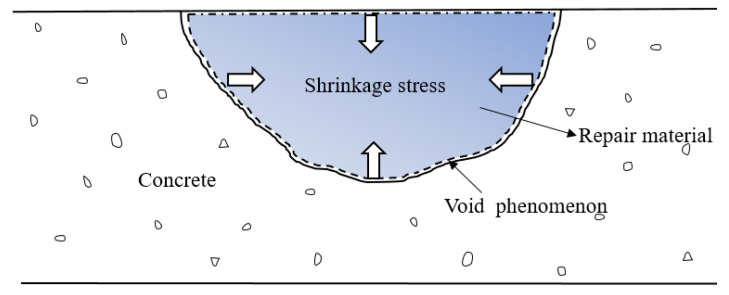
Shrinkage interface between the repair material and concrete.

Comparing the bond strength of the repair material without and with PVAc + styrene, we can surmise that after adding PVAc + styrene, the bond strength of repair material decreased, especially in the early stage. This may be because the addition of PVAc + styrene causes a significant decrease in shrinkage stress, leading to the reduction of local void phenomenon at the bonding interface, as shown in Figure 9, and the improvement of bond strength. However, at the same time, micropores generated inside the material, as shown in SEM micrographs, reduce the bond strength, and the degree of reduction is greater than the degree of improvement, so the overall bonding performance of the material becomes worse.

As a material for repairing cracks, it should be able to resist the bending moment without fracture within a certain range. To determine this fracture resistance, this experiment tested the effect of different proportions of PVAc + styrene on the bending strength of MMA-based repair material; the results obtained are shown in Table 4.

It can be seen from the Table 4 that with the extension of the curing time, the bending strength of the material gradually increased and the difference between the bending strength at 7 d and 28 d was not significant. After the addition of additives, the change trend of the bending strength became complex, but most of the bending strengths increased to a certain extent. When the proportion of PVAc + styrene was 14%, the bending strength of the material could reach 32.81 MPa. This is mainly because after the admixture of additives, during the polymerization reaction, the free radicals decomposed by the initiator activate not only the MMA molecules but also the styrene molecules, so the growth rate of PMMA main chain slows down. At the same time, during the curing process, the steric hindrance generated would reduce the reaction rate and the heat release, so as to avoid the internal scorching of the repair material due to the large amount of exotherm, which has little impact on the bending strength. Therefore, the incorporation of low-shrinkage additives results in a substantial increase in the bending strength of the repair material.

**Figure 10:**
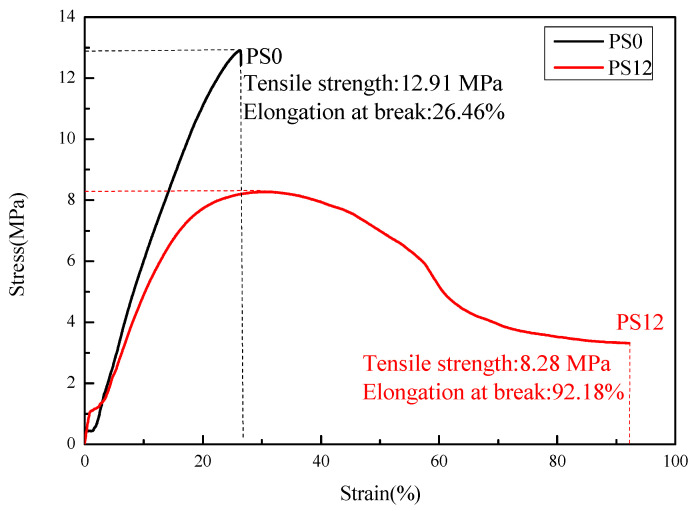
Tensile stress–strain curve of the MMA-based repair material.

Tensile strength is one of the most important mechanical properties and reflects the resistance of the material to the lateral forces after repair engineering. Two repair materials, PS0 and PS12, were used to make tensile specimens and conduct the experiments, and the tensile stress–strain curves were plotted as shown in Figure 10.

From the curves, it can be discerned that the specimen without PVAc + styrene was pulled out with a tensile load up to 258.26 N, the corresponding elongation at break was 26.46%, the tensile strength was 12.91 MPa, and the elastic modulus was 70.79 MPa. After the addition of 12% PVAc + styrene, the material broke at a tensile load of 165.57 N, with an elongation at break of 92.18%, a tensile strength of 8.28 MPa and an elastic modulus of 54.35 MPa. As seen in Figure 10, the stress–strain curves of the repair materials were basically smooth, which is consistent with the characteristics of plastic materials. When PVAc + styrene was not incorporated, the deformation of the material after yielding was small. At this time, the elongation at the break of the repair material was small, and the tensile strength and elastic modulus were large, demonstrating hard and strong characteristics. After the incorporation of 12% PVAc + styrene, the deformation of the material after yielding was greater, the elongation at the break increased significantly, with improved fracture toughness of the material.

It can be seen that the addition of 12% PVAc + styrene reduced the elastic modulus of MMA-based repair material. Generally, the lower elastic modulus corresponded to the smaller shrinkage stress. This occurred because the lower elastic modulus can make the stress dissipate better in the polymerization process, so the shrinkage stress was 87.4% lower than that of the material without low-shrinkage additives, as shown in Table 2. Because the generated two-phase system had poor compatibility and was not conducive to stress transmission, the tensile strength of the repair material added with low-shrinkage additives decreased, and the existence of micropores and steric hindrance also affected it. Although the tensile strength of the material decreased, its elongation at break increased significantly, indicating that it would not cause brittle fracture immediately when subjected to tension.

From the measurements of shrinkage, viscosity and mechanical properties, it can be concluded that PVAc and styrene as a low-shrinkage additives had a considerable effect on the overall performance of the MMA-based repair material. Based on the results of this study, it was found that the addition of low-shrinkage additives had a favorable effect on the shrinkage and shrinkage stress of the repair material but a more variable effect on the mechanical properties. This is because the two-phase structure and the formation of micropores compensate for the volume shrinkage and reduce the shrinkage stress, but the presence of micropores also has an adverse effect on some mechanical properties of the material. It is possible to obtain MMA-based repair material with specific requirements by varying the content of low-shrinkage additives to repair concrete microcracks.

## 4. Conclusions

The main conclusions are as follows:

1. The addition of the low-shrinkage additives of PVAc and styrene significantly reduced the volume shrinkage and shrinkage stress of the MMA-based repair material and delayed the polymerization reaction to some extent. When the content of PVAc + styrene was 12%, the volume shrinkage of the repair material was the lowest, only 4.78%, and the shrinkage stress decreased by 87.4%.

2. During the polymerization process, styrene and MMA undergo copolymerization, which reduces the volume shrinkage to a certain extent through a steric hindrance effect. The polystyrene generated during the polymerization counteracts the curing shrinkage mainly by thermal expansion. The formation of micropores at the interface between the continuous phase of MMA and the dispersed phase of PVAc also offsets part of the volume shrinkage.

3. After the addition of low-shrinkage additives, the bond strength of the MMA-based repair material was relatively low after 3 d, and the fracture surfaces all appeared at the bonding interface between the repair material and the OPC mortar. At 28 d, the specimens all had fractures inside the OPC mortar in testing, showing a good bonding performance.

4. PVAc + styrene improved the bending strength of most of the repair materials in the ratios investigated in this study; when the proportion was 12%, the 28 d bending strength of the material reached 28.04 MPa, and the elongation at break and the tensile strength of the MMA-based repair material were 92.18% and 8.28 MPa, respectively.

## Figures and Tables

**Table 1:** Raw material ratio of MMA-based repair material (wt%).

NO.	PVAc + Styrene/%	MMA	BPO	DOP	DMA	PVAc	Styrene
PS0	0	76.22	0.46	22.86	0.46	0	0
PS10	10	68.60	0.41	20.58	0.41	7.00	3.00
PS11	11	67.83	0.41	20.35	0.41	7.70	3.30
PS12	12	67.08	0.40	20.12	0.40	8.40	3.60
PS13	13	66.32	0.39	19.90	0.39	9.10	3.90
PS14	14	65.56	0.39	19.66	0.39	9.80	4.20

**Table 2:** Shrinkage results of MMA-based materials with different PVAc + styrene contents.

NO.	Before Curing			After Curing			Volume Shrinkage/%	Shrinkage Stress/N
	Mass/g	Volume /mL	Density /g·mL^−1^	Mass/g	Volume /mL	Density /g·mL^−1^		
PS0	3.75	4.30	0.87	3.68	3.44	1.07	22.67	−5.08 *
PS10	4.17	4.10	1.02	3.88	3.55	1.09	7.46	−2.23
PS11	4.20	4.05	1.04	4.16	3.75	1.11	6.97	−0.78
PS12	4.19	4.02	1.04	4.03	3.69	1.09	4.78	−0.64
PS13	4.26	4.29	0.99	4.19	3.83	1.09	10.17	−0.71
PS14	4.18	4.39	0.95	3.98	3.65	1.09	14.51	−2.95

* Negative stress values indicate shrinkage.

**Table 3:** Effect of PVAc + styrene contents on bond strength of MMA-based repair materials.

No.	3 d		7 d		28 d	
	Bond Strength/MPa	Fracture Location	Bond Strength/MPa	Fracture Location	Bond Strength/MPa	Fracture Location
PS0	>4.62 *	M *	>5.32	M	>5.51	M
PS10	2.60	I *	3.90	I	>4.21	M
PS11	3.84	I	>4.07	M	>4.39	M
PS12	2.93	I	>4.04	M	>4.10	M
PS13	2.83	I	>3.97	M	>4.13	M
PS14	2.63	I	3.83	I	>4.03	M

* “M” represents the fracture surface occurring inside the mortar specimen. “I” represents the fracture surface appearing at the bonding interface. “>” represents a bond strength greater than the obtained flexural strength.

**Table 4:** Bending strength of the repair materials.

No.		PS0	PS10	PS11	PS12	PS13	PS14
Bending strength /MPa	3d	19.69	21.73	22.97	20.74	18.78	24.28
	7d	25.93	31.38	30.25	26.25	23.79	31.11
	28d	27.52	31.48	31.56	28.04	26.17	32.81

## Data Availability

All data in this article are listed in this paper. The data presented in this study are available on request from the corresponding author.
